# CT-based AI framework leveraging multi-scale features for predicting pathological grade and Ki67 index in clear cell renal cell carcinoma: a multicenter study

**DOI:** 10.1186/s13244-025-01980-0

**Published:** 2025-05-14

**Authors:** Huancheng Yang, Yueyue Zhang, Fan Li, Weihao Liu, Haoyang Zeng, Haoyuan Yuan, Zixi Ye, Zexin Huang, Yangguang Yuan, Ye Xiang, Kai Wu, Hanlin Liu

**Affiliations:** 1 Department of Radiology, The Sixth Affiliated Hospital of Sun Yat-sen University, Guangzhou, China; 2 Department of Radiology, The Third Affiliated Hospital of Shenzhen University (Luohu Hospital Group), Shenzhen, China; 3 Department of Radiology, The Second Affiliated Hospital of Soochow University, Suzhou, China; 4 Department of Radiology, The Third Hospital of Mianyang, Sichuan Mental Health Center, Mianyang, China; 5 Shantou University Medical College, Shantou University, Shantou, China; 6 Department of Radiology, Shenzhen Luohu District Traditional Chinese Medicine Hospital (Luohu Hospital Group), Shenzhen, China; 7 Department of Radiology, Leshan Hospital, Chengdu University of Traditional Chinese Medicine, Leshan, China

**Keywords:** Clear cell renal cell carcinoma, Machine learning, Ki67, WHO classification, Renal cell carcinoma pathology/grading

## Abstract

**Purpose:**

To explore whether a CT-based AI framework, leveraging multi-scale features, can offer a non-invasive approach to accurately predict pathological grade and Ki67 index in clear cell renal cell carcinoma (ccRCC).

**Methods:**

In this multicenter retrospective study, a total of 1073 pathologically confirmed ccRCC patients from seven cohorts were split into internal cohorts (training and validation sets) and an external test set. The AI framework comprised an image processor, a 3D-kidney and tumor segmentation model by 3D-UNet, a multi-scale features extractor built upon unsupervised learning, and a multi-task classifier utilizing XGBoost. A quantitative model interpretation technique, known as SHapley Additive exPlanations (SHAP), was employed to explore the contribution of multi-scale features.

**Results:**

The 3D-UNet model showed excellent performance in segmenting both the kidney and tumor regions, with Dice coefficients exceeding 0.92. The proposed multi-scale features model exhibited strong predictive capability for pathological grading and Ki67 index, with AUROC values of 0.84 and 0.87, respectively, in the internal validation set, and 0.82 and 0.82, respectively, in the external test set. The SHAP results demonstrated that features from radiomics, the 3D Auto-Encoder, and dimensionality reduction all made significant contributions to both prediction tasks.

**Conclusions:**

The proposed AI framework, leveraging multi-scale features, accurately predicts the pathological grade and Ki67 index of ccRCC.

**Critical relevance statement:**

The CT-based AI framework leveraging multi-scale features offers a promising avenue for accurately predicting the pathological grade and Ki67 index of ccRCC preoperatively, indicating a direction for non-invasive assessment.

**Key Points:**

- Non-invasively determining pathological grade and Ki67 index in ccRCC could guide treatment decisions.
- The AI framework integrates segmentation, classification, and model interpretation, enabling fully automated analysis.
- The AI framework enables non-invasive preoperative detection of high-risk tumors, assisting clinical decision-making.

**Graphical Abstract:**

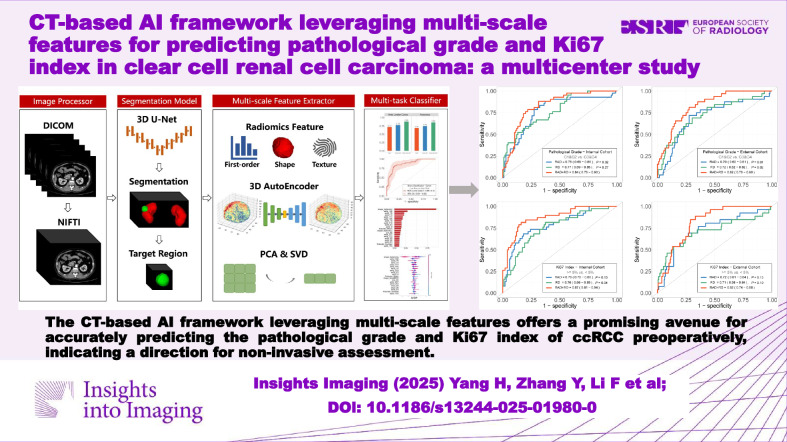

## Introduction

Renal cell carcinoma (RCC) stands as one of the most prevailing urological malignancies, wherein a striking 70–80% of cases are attributed to the classification of clear cell renal cell carcinoma (ccRCC) [1]. Over time, ccRCC has experienced a rise in prevalence, adorning itself with the title of the harbinger of the greatest number of metastatic events and fatalities among all the diverse subtypes of RCC [2]. In 2016, the World Health Organization/International Society of Urological Pathology (WHO/ISUP) classified tumors with nuclear grades I and II as low-grade, and those with nuclear grades III and IV as high-grade [3]. High-grade ccRCC displays greater aggressiveness and leads to unfavorable patient prognoses [2]. Ki67 is a molecular marker used to determine cell proliferation and is expressed exclusively during the G1, S, G2, and M phases of the cell cycle, but not in the G0 phase. In typical clinical practice, the standard procedure for determining the Ki67 index involves employing immunohistochemistry (IHC) to stain tissue samples obtained from renal tumors excised during surgery [2]. Its strong correlation with cell proliferation and simultaneous expression with other well-established proliferation markers suggests its crucial role in cell division. Multiple studies have reported a significant association between high Ki67 expression and poor prognosis in ccRCC [4–8]. Currently, histological evidence of RCC is typically obtained from excised specimens after surgery, whereas when preoperative pathological evidence is required, it usually relies on percutaneous renal biopsy. However, this method carries risks, including bleeding and infection [9]. Furthermore, this invasive procedure poses a myriad of challenges, as it fails to comprehensively sample every facet of the tumor and lacks the capability for repeated sampling, thus impeding a comprehensive reflection of the tumor’s heterogeneity and precise differentiation of its pathological grade, as well as impeding the assurance of accurate immunohistochemical results for Ki67 [10]. As a result, an imperative need arises for a non-invasive and reproducible approach to assess the tumor’s pathological grade and Ki67 index, unrestricted by temporal or spatial limitations in tissue sampling.

In recent times, artificial intelligence (AI) algorithms have exhibited tremendous potential in the realm of imaging analysis for ccRCC. Zheng et al [11] devised a radiomics scoring framework rooted in CT imaging, adept at predicting the grade of ccRCC. Cui et al [12] discerned that the predictive prowess of multiphase CT machine learning surpasses its single-phase counterpart in grade prognostication. Furthermore, Demirjian et al [13] achieved successful ccRCC TNM staging by leveraging radiomic signatures extracted from multiphase CT scans. However, these inquiries are not exempt from limitations that necessitate thoughtful consideration. Firstly, the models are contingent upon the adept manual annotation of regions of interest (ROI) by specialists, a practice that becomes impractical in various scenarios. Secondly, these studies frequently depend on limited data from single-center or dual-center sources with similar machine characteristics, resulting in poor generalization performance of the trained models in other centers and posing challenges for translation into clinical applications. Thirdly, the cryptic nature of deep learning algorithms, often denoted as the “black box,” complicates the interpretation of the decision-making process within the model, posing a challenging endeavor and eliciting concerns among clinicians regarding its application.

To the best of our knowledge, there is currently no literature reporting on a multitasking AI framework for the fully automated prediction of both the pathological grade and Ki67 index in ccRCC. In our investigation, we formulated a new automated analysis framework, leveraging a multi-center dataset and multi-scale features, to proficiently discern the ccRCC’s pathological grade (G1&G2 vs G3&G4) and Ki67 index (≥ 5% vs < 5%). The AI framework seamlessly amalgamated radiomics features, 3D Auto-Encoder features, and dimension reduction features as inputs. Furthermore, we harnessed the power of Shapley additive explanations (SHAP) values to scrutinize the influence of each extracted feature on model decisions, thus illuminating the intricacies of the decision-making process.

## Materials and methods

### Participant cohorts and data selection

This is a retrospective investigation conducted on cohorts derived from multiple centers. A total of 1073 individuals with pathologically confirmed ccRCC were divided into the internal dataset (*n* = 857) and the external testing dataset (*n* = 216). The internal dataset comprises participants who underwent nephrectomy sourced from five publicly accessible datasets (CPTCA-CCRCC, TCGA-KIRC, TCGA-KIRP, TCGA-KICH, and C4KC-KiT obtained from the Cancer Imaging Archive), as well as five local hospitals spanning the timeframe from 2017 to 2023. The external testing set consisted of individuals who underwent nephrectomy at a different local hospital between 2017 and 2023. Informed consent documentation was waived given that this retrospective analysis employed de-identified clinical data and images. This study adheres to the STROCSS standards [14]. Ethical endorsement for this study was obtained from the local institutional review board (approval no. 2023-LHQRMYY-KYLL-55).

**Fig. 1:**
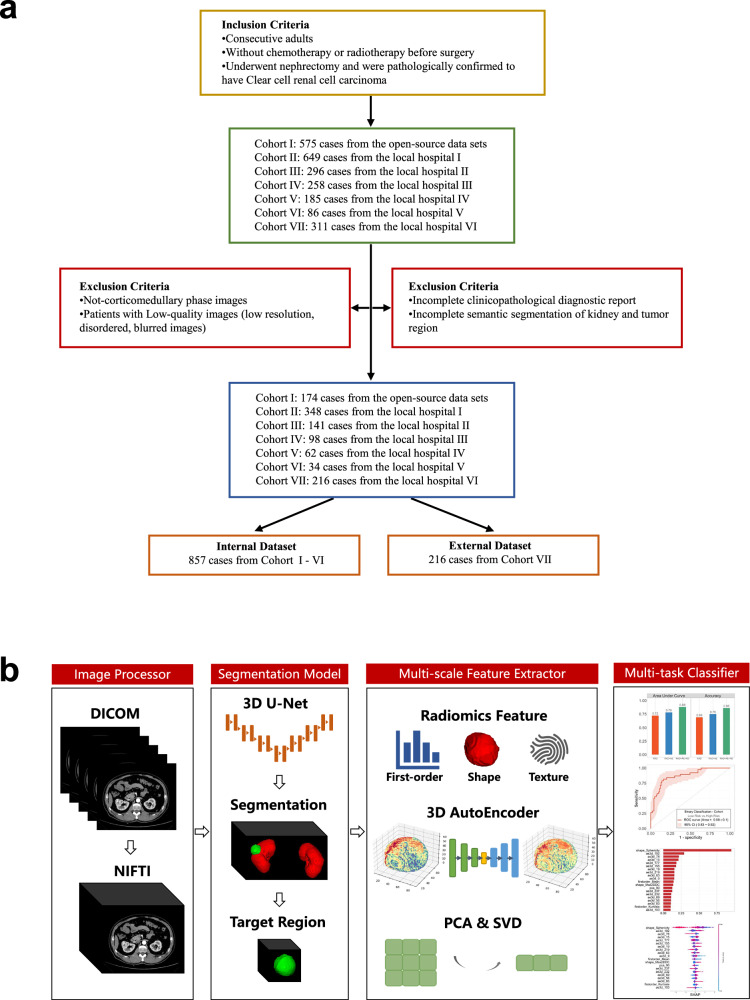
The AI framework. **a** Workflow schema. Showing the inclusion and exclusion criteria for patient selection for open-source data sets (CPTCA-CCRCC, TCGA-KIRC, TCGA-KIRP, TCGA-KICH, and C4KC-KiT from The Cancer Imaging Archive) and six different local hospitals. **b** Overall analysis framework. Our framework takes an original CT image (DICOM) as input. The proposed AI framework contains the following modules: an image processor, a 3D-tumor segmentation model based on 3D-UNet, a multi-scale features extractor, and a multi-task classifier

In this investigation, we adhered to the precisely defined inclusion and exclusion criteria, as detailed in Fig. 1a. The inclusion criteria comprised the following: 1. consecutively enrolled adult individuals, 2. those who had not undergone any prior chemotherapy or radiotherapy before their surgical procedure, 3. individuals who underwent nephrectomy and received a histopathological confirmation of having ccRCC, and 4. corticomedullary phase (CMP) CT was performed prior to surgery. On the contrary, the exclusion criteria encompassed the following: 1. patients with low-quality images (characterized by low resolution, disorderliness, or blurriness), 2. incomplete clinicopathological diagnostic reports, and 3. incomplete semantic segmentation of the kidney and tumor regions.

### Image processing and segmentation model

Detailed machine scanning parameters for the acquisition of CT images for each cohort can be found in the supplementary materials and Table S1. The complete analytical process of this study, specifically, the AI framework we devised, is illustrated in Fig. 1b. In this study, we availed ourselves of the dcm2niix tool to fashion an image processor, which seamlessly transmutes DICOM format images into the NIFTI format [15]. Subsequently, two radiologists with over 10 years of experience, along with two trained medical students, meticulously delineated the ROI for the kidneys and kidney cancer in the CMP CT images. Finally, a senior radiologist with more than two decades of expertise, ensured the exactness of the tumor and kidney boundary delineations.

Drawing inspiration from the manual segmentation results and the three-dimensional imagery, we contrived an automated segmentation model for both kidneys and kidney cancer, harnessing a sophisticated 3D-UNet-based network [16]. The fundamental principle of 3D-UNet involves utilizing a convolutional neural network for simultaneous feature extraction and segmentation, seamlessly integrating feature maps at various scales through skip connections.

### Multi-scale feature extractor

In this study, we developed a multi-scale feature extractor aimed at comprehensively capturing the anatomical complexities. This feature extractor integrates radiomics features, deep learning-based nonlinear dimensionality reduction features from a 3D Auto-Encoder, and linear dimensionality reduction features derived from principal component analysis (PCA) and singular value decomposition (SVD). First, we extracted 100 radiomics features using PyRadiomics (v3.0.1), which include first-order statistics, shape features, and texture characteristics, all related to kidney cancer. These features quantify the heterogeneity of the tumor from various perspectives, providing foundational information for subsequent analysis. Next, we designed a 3D Auto-Encoder to extract 256 deep learning-based nonlinear dimensionality reduction features. This Auto-Encoder captures deep features of the tumor region in an unsupervised manner. The model consists of six layers, with the encoder containing three 3D convolutional layers that progressively compress the input data into low-dimensional latent representations. The decoder includes three corresponding 3D deconvolution layers that reconstruct the original input from the latent representations. During training, we used Mean Squared Error as the reconstruction loss function to measure the difference between predicted and true values, and the model was trained for 500 epochs using the Adam optimizer. Finally, we performed matrix linear dimensionality reduction on the segmented voxels of the CT images. Specifically, based on the ROI, we cropped a 64 × 64 × 64 voxel region that included only the tumor and kidney tissue, filling the blank areas of the cropped image with the minimum pixel value. Then, using scikit-learn, we employed both SVD and PCA to reduce the dimensionality of the cropped region to 64 and 256, respectively, extracting linear features that represent the original voxel matrix information.

In summary, for each CT image, our multi-scale feature extractor extracted a total of 676 features, including radiomics features, nonlinear dimensionality reduction features, and linear dimensionality reduction features.

### Multi-task classifier

A total of 857 cases from the internal cohort were judiciously stratified and split into a training set (80%, 686 cases) and a validation set (20%, 171 cases). Moreover, 216 cases from the external cohort were enlisted in the external testing set to evaluate the model’s generalization capability. To enhance training efficiency, we directly chose the highly popular XGBoost (XB) algorithm to build classification models for the two tasks of pathological grading (G1&G2 vs G3&G4) and Ki67 index (≥ 5% vs < 5%). During the model training process, we optimized the hyperparameters using Grid Search. Furthermore, we compared the performance of classification models using radiomics features, newly designed dimensionality reduction features (3D Auto-Encoder, PCA, and SVD), and multi-scale features (fusion of radiomics and dimensionality reduction features) as inputs, respectively. Finally, to confirm the robust performance of XB, we utilized three other machine learning classification algorithms—random forest (RF), LightGBM (LG), and CatBoost (CB)—to compare the performance of these four algorithms on the two prediction tasks.

### Model explaining and statistical analysis

SHAP [17, 18] is an algorithm intricately crafted to illuminate the decisions orchestrated by machine learning models. Anchored in the profound concept of SHAP values derived from cooperative game theory, its primary aim is to precisely quantify the contribution of each feature to the model’s output. In our endeavor to evaluate the influence of CT image features on the model predictions, we computed the SHAP values for each feature, dissecting the model’s decisions into the nuanced influences of individual features for each sample. All of these experiments and statistical analyses were conducted using Python (v3.8) and R (v3.6.3). Statistical significance was deemed relevant when the *p*-value was less than 0.05. The uncertainty of estimates, such as accuracy and area under the receiver operating characteristic (AUROC), was quantified within a 95% confidence interval.

## Results

### Patient and dataset characteristics

CT images with clinical, pathological, and IHC information were gathered and curated from open-source cohorts, as well as local cohorts. Following a rigorous image screening process (Fig. 1a), 1073 participants (comprising 465 females and 608 males) were selected for subsequent analysis. The foundational and clinical particulars of these participants are showcased in Table 1. The mean age (with standard deviation) was observed to be 56.3 (13.3) years. For the final evaluation of our pioneering model, the external testing set consisted of 216 cases, thoughtfully chosen for this purpose. A depiction of the AI framework is shown in Fig. 1b.

**Table 1:** Patient demographics, surgical data, and pathologic characteristics for the internal and external cohort

Characteristic	Internal cohort	External cohort
Participants (1073)	857	216
Age (year)	56.34 ± 12.53	55.79 ± 12.56
Sex		
Female	380 (44.34%)	85 (39.35%)
Male	477 (55.66%)	131 (60.65%)
Nephrectomy		
Partial nephrectomy	524 (61.14%)	127 (58.80%)
Radical nephrectomy	333 (38.86%)	89 (41.20%)
Not available	0 (0%)	0 (0%)
Pathologic tumor grade		
Low grade (G1/G2)	523 (61.03%)	184 (85.19%)
High grade (G3/G4)	210 (24.50%)	32 (14.81%)
Not available	124 (14.47%)	0 (0%)
IHC (Ki67)		
Low Ki67 (< 5%)	496 (57.88%)	187 (86.57%)
High Ki67 (≥ 5%)	187 (21.82%)	29 (13.43%)
Not available	174 (20.30%)	0 (0%)
Radiomics features		
Shape_Maximum3DDiameter	64.81 ± 46.64	57.85 ± 33.79
Firstorder_10Percentile	32.28 ± 26.49	19.95 ± 29.12
Firstorder_90Percentile	163.36 ± 57.78	136.67 ± 48.06

### Kidney and tumor region segmentation of the 3D-UNet model

**Fig. 2:**
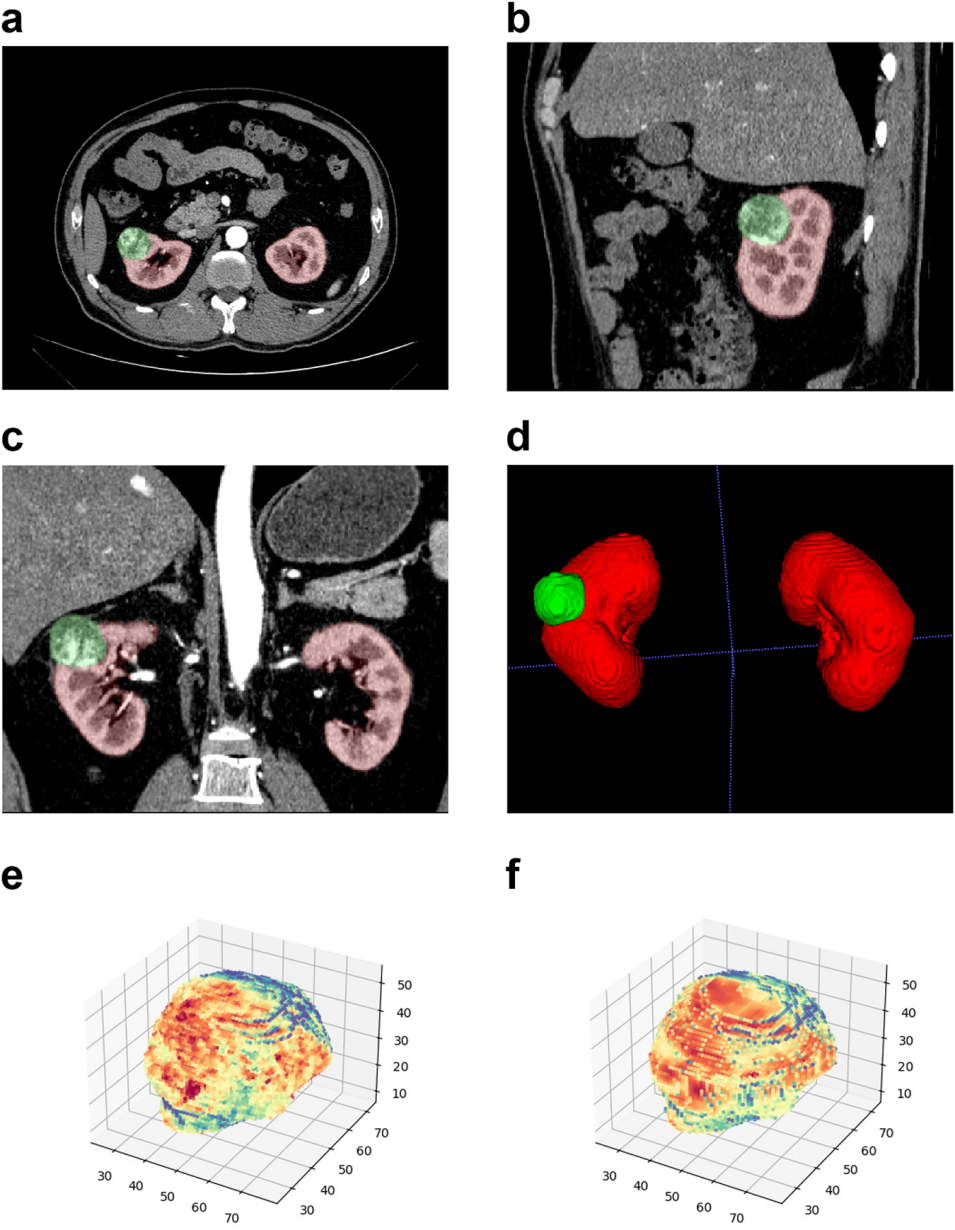
Predictive segmentation and 3D Auto-Encoder result of one case in the external test set. In the segmentation results, the red and green color represents the kidney and tumor, respectively. **a** Axial segmented image. **b** Sagittal segmented image. **c** Coronal segmented image. **d** 3D segmented image. The tumor target region is the input (**e**) and output (**f**) of the 3D Auto-Encoder

Employing the 3D-UNet architecture as its bedrock, we trained a multi-label semantic segmentation model, adeptly designed for the automated segmentation of the kidney and tumor regions within CT images. This model exhibits excellent, accurate tumor segmentation on the vast majority of data, with a dice coefficient greater than 0.92. In 216 patients of the testing set, the mean dice coefficient with standard deviation was 0.979 (0.030) for the kidney and 0.924 (0.094) for the tumor region (Fig. S1). Figure 2a–d showcases the predictive segmentation of a case from the testing set, comprising the Axial segmented image (a), the sagittal segmented image (b), the coronal segmented image (c), and the 3D segmented image (d).

### Multi-scale features solution enhances the best capability

The 3D tumor images were automatically cropped to the matrix ROI, and 676 image features were extracted using our designed multi-scale feature extractor. Figure 2e, f displays the tumor target region, presenting it as the input and output of the 3D Auto-Encoder. A preliminary experiment was conducted to ascertain the optimal combinations of multi-scale features.

**Fig. 3:**
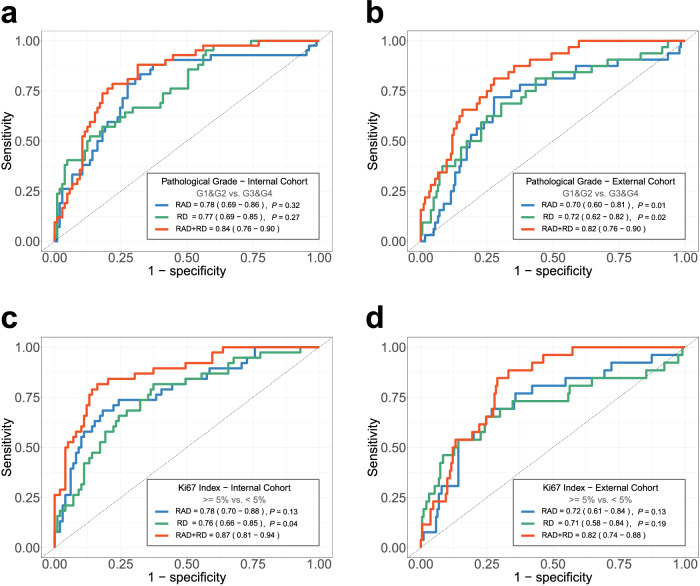
Predictive performances of diverse feature extraction solutions in the internal and external cohorts (**a**–**d**). XG, XGBoost; RAD, radiomics; RD, dimensionality reduction. ROC test (bootstrap method) was used for statistical comparison between RAD and RD, respectively, with RAD + RD. Plots show the ROC curves of three feature extraction solutions, in the classification tasks of pathological grade (G1&G2 vs G3&G4) and Ki67 index (≥ 5% vs < 5%) (**a**–**d**), respectively, in the internal and external cohorts

In classification tasks, the strategy of multi-scale feature fusion significantly enhanced performance. When distinguishing pathological grade (G1&G2 vs G3&G4), using radiomics features, dimensionality reduction features, and multi-scale features as inputs in the internal cohort yielded AUROC values of 0.78, 0.77, and 0.84, respectively (Fig. 3a), while in the external cohort, the corresponding AUROC values were 0.70, 0.72, and 0.82 (Fig. 3b). In the task of predicting the Ki67 index (≥ 5% vs < 5%), using radiomics features, dimensionality reduction features, and multi-scale features as inputs in the internal cohort resulted in AUROC values of 0.78, 0.76, and 0.87, respectively (Fig. 3c), whereas in the external cohort, the corresponding AUROC values were 0.72, 0.71, and 0.82 (Fig. 3d).

### The AI framework presents robust analytical performance

**Fig. 4:**
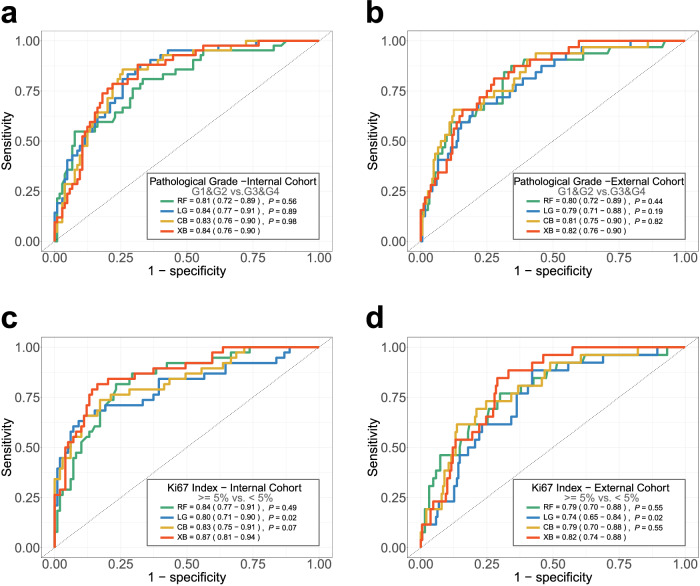
Predictive performances of diverse machine learning algorithms in the internal and external cohorts (**a**–**d**). ROC test (bootstrap method) was used for statistical comparison between RF, LG, and CB algorithms, respectively, with the Extreme XGBoost (XB) algorithm. Plots show the ROC curves of RF, LG, CB, and XB algorithms, in the classification tasks of pathological grade (G1&G2 vs G3&G4) and Ki67 index (≥ 5% vs < 5%), respectively, in the internal and external cohorts

To verify the robust analytical capability of our algorithms, we compared the model performance trained by the RF, LG, CB, and XB algorithms, and found that the last one had the best performance in all classification tasks. Figure 4a–d demonstrates that none of the machine learning algorithms achieved the same level of AUROC as the XB algorithm in two classification tasks. Additionally, the receiver operating characteristic (ROC) test identified statistically significant differences in the pathological grade (G1&G2 vs G3&G4), and Ki67 index (≥ 5% vs < 5%) classification. Therefore, we selected the XB algorithms as the prediction model for this study. In internal validation, the optimal models achieved AUROC values of 0.84 for pathological grade classification (G1&G2 vs G3&G4) and 0.87 for Ki67 index classification (≥ 5% vs < 5%) (Fig. 4a, c). As we ventured into the external testing set, the AUROC values presented themselves as 0.82 and 0.82, respectively, alluringly showcased in Fig. 4b, d.

The detailed performance parameters of the model are presented in Table S2. The results demonstrate that the model significantly improves sensitivity and accuracy while maintaining high specificity.

### Evaluating feature contributions through SHAP values

To delve into the influence of individual features on model prediction, we computed the SHAP values for each feature with respect to each sample. Based on these SHAP values, the top 20 contributors for predicting pathological grade (G1&G2 vs G3&G4 and Ki67 index (≥ 5% vs < 5%) were eloquently portrayed on bee-swarm plots and bar plots. The bee-swarm plot artfully displays the SHAP values and feature values across the original dataset, with the color of each dot gracefully indicating the magnitude of its corresponding eigenvalue. Redder dots signify larger eigenvalues, while bluer dots indicate smaller eigenvalues. Positive SHAP values signify a higher probability associated with the respective prediction.

**Fig. 5:**
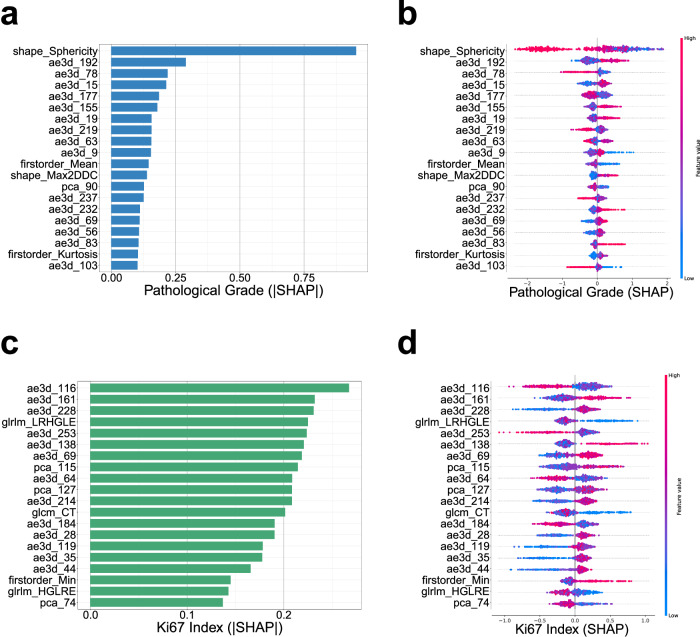
Ranking of SHAP values for the explanation of the proposed model's decision-making in the training set (**a**–**d**). Bar-plots and bee-plots display the top-20 drivers’ features SHAP values in pathological grade (G1&G2 vs G3&G4) and Ki67 index (≥ 5% vs < 5%), respectively

Regarding the bar plots, in Fig. 5a, b, the radiomics feature “shape Sphericity” played a preeminent role in the model’s decision-making for pathological grade (G1&G2 vs G3&G4), while 3D Auto-Encoder features (such as ae3d_192, ae3d_78, ae3d_15, ae3d_177, and ae3d_155) also showcased substantial contributions to the model’s decision. In Fig. 5c, d, features extracted by the 3D Auto-Encoder (e.g., ae3d_116, ae3d_161, ae3d_228, ae3d_253, and ae3d_138), as well as radiomics features (e.g., glrlm_LRHGLE), made significant contributions to the model’s decisions regarding Ki67 index predictions (≥ 5% vs < 5%). Furthermore, features extracted by dimensionality reduction (such as pca_115 and pca_127) also participated in the model’s prediction.

## Discussion

In our study, we established a comprehensive thin-slice CT image database with 1073 ccRCC patients from seven medical centers. We devised an automated framework for lesion segmentation, multi-scale feature extraction, and multi-task prediction, enabling non-invasive preoperative assessment of pathological grade (G1&G2 vs G3&G4) and Ki67 index (≥ 5% vs < 5%). This AI framework integrates radiomic, 3D Auto-Encoder, and dimension reduction features, providing more comprehensive image analysis. We computed SHAP values for each feature, revealing the decision-making process and addressing black-box issues in machine learning. Our framework offers a cutting-edge solution for preoperative assessment and enhances understanding of AI model decision processes.

The pathological grade of ccRCC significantly impacts patient prognosis, with higher grades indicating more aggressive tumors and worse outcomes [2]. In the past, the widely accepted Fuhrman grading system categorized ccRCC tumors into four tiers based on pathological characteristics. However, in modern clinical practice, the WHO/ISUP grading system has replaced the Fuhrman system, providing a refined classification that predicts cancer-specific mortality and promotes diagnostic consistency [19]. Ki67, a common molecular marker for assessing tumor proliferation, plays a crucial role in cancer prognosis. This nuclear antigen is present during various phases of the cell cycle in all cycling human cells, and its expression levels are linked to the prognosis of different neoplasms, including kidney cancer [2]. A large-scale study by Tollefson et al revealed that increased Ki67 expression in RCC patients was associated with a 68% higher risk of mortality and correlated strongly with adverse pathological features [20]. Other studies have also confirmed the link between elevated Ki67 expression and reduced cancer-specific survival [4, 21]. However, the current conventional method for preoperative assessment of pathological grade and Ki67 labeling index involves minimally invasive percutaneous renal biopsy, which has issues with sampling bias that fail to fully capture tumor heterogeneity and face challenges such as non-repeatability and complications like tumor metastasis. Therefore, it fails to fully capture tumor heterogeneity and accurately determine pathological nuclear grade and precise Ki67 index.

As a nascent methodology, CT radiomics empowers the quantification of tumor heterogeneity through the analysis of the spatial distribution of imaging voxels and variations in signal intensity [22]. This groundbreaking approach enables the discernment of subtle dissimilarities in intensity distribution within medical images, facilitating the non-invasive prognostication of tumor pathological outcomes with extraordinary precision [23, 24]. Nevertheless, there exists a rather limited body of research concerning the implementation of CT radiomics to predict pathological outcomes in ccRCC [11, 13, 25]. Most of these investigations necessitate manual segmentation of the ROI and fall short in comparability to our automated segmentation model. In stark contrast to previous inquiries that exclusively extracted 2D features from the tumor center, our study undertook a more all-encompassing extraction of image features, encompassing radiomics, predicated on 3D ROI. Consequently, our multi-scale features furnish a more exhaustive assessment of tumor heterogeneity.

Recently, the application of machine learning algorithms has demonstrated promise in the scrutiny of renal tumor CT imaging [26, 27]. However, in some previous investigations, machine learning models were introduced without sufficient expository research or transparency, thus assuming the semblance of ‘black boxes’ [28]. To rectify this concern, we conducted a quantitative analysis to scrutinize the correlation between multi-scale features and model decisions, employing SHAP values. In comparison to other interpretability methodologies, such as class activation map [29], SHAP value possesses the precision to gauge the influence of each feature both individually and on the entire dataset. This endows a more compelling evaluation of the model’s decision-making process, encompassing the rationale behind misclassifications. From Fig. 5, we discern that the radiomics feature, shape Sphericity, contributed most significantly to the prediction of low- vs high-pathological grade, which aligns with Yang et al's study [30], likely signifying disparities in the physical growth of pathological grade (G1&G2 vs G3&G4). In the prediction of the Ki67 index (≥ 5% vs < 5%), refined features obtained through 3D Auto-Encoder and PCA&SVD dominated the entire prognostication. These features serve as an abstraction or mathematical representation of the tumor region, imperceptible to the visual eye, and potentially offering a means to evaluate tumor grade via CT. These findings advocate that the multi-scale features assume a complementary role, heightening model performance in predicting low-grade vs high-grade pathological grade and low vs high Ki67 index.

Compared to other studies [11–13], our established medical imaging database features a larger and higher-quality sample size. Our database includes thin-slice CT data from 1073 ccRCC patients, collected from seven major medical centers. The framework we propose enables fully automated analysis, requiring only preoperative CT input. Importantly, our database surpasses similar studies in scale and quality [11–13]. Our framework allows for an interpretable analysis of model decision-making processes. Ultimately, our model demonstrates superior performance. In external test cohorts, our model achieved an AUROC of 0.82 for predicting ccRCC’s pathological grade and Ki67 index. These aspects further highlight the potential of AI models to streamline physicians’ workflow in the future.

In this study, certain limitations must be acknowledged. Firstly, the current framework doesn’t completely replace preoperative invasive pathological biopsies for ccRCC patients but serves as a complement for clinicians in preoperative decision-making. Secondly, benign renal lesions like angiomyolipoma and renal adenoma weren’t included in this investigation, and the model’s predictions for benign conditions weren’t scrutinized. Finally, our focus was on predicting the pathological grade and Ki67 index of ccRCC through retrospective medical records, introducing uncertainty regarding whether this approach can improve patient outcomes. Hence, prolonged follow-up and further prospective inquiries are crucial in the future.

In summary, we’ve presented an automated, non-invasive multi-scale features framework for classifying pathological grade and Ki67 index, leveraging a diverse dataset from multiple centers. Furthermore, our study confirmed a significant enhancement in the model’s overall performance with multi-scale feature integration. Additionally, a quantitative analysis of extracted features’ impact on each model decision was conducted using SHAP values, shedding light on the decision-making process. We strongly believe this research provides invaluable insights to inform clinical decisions, harnessing the potential of medical images and machine learning techniques.

## Supplementary information

ELECTRONIC SUPPLEMENTARY MATERIAL

## Data Availability

The original images and data used in this study are available from the corresponding author by request.

## Abbreviations

AI: Artificial intelligence
AUROC: Area under the receiver operating characteristic
ccRCC: Clear cell renal cell carcinoma
CMP: Corticomedullary phase
ROC: Receiver operating characteristic
ROI: Regions of interest
SHAP: Shapley additive explanations
