# Supplementary material for: CT-based AI framework leveraging multi-scale features for predicting pathological grade and Ki67 index in clear cell renal cell carcinoma: a multicenter study
Source: Insights Imaging. 2025 May 14;16:102. doi: 10.1186/s13244-025-01980-0 (PMC12078187; doi:10.1186/s13244-025-01980-0)
Supplement: Supplementary file 1 — ELECTRONIC SUPPLEMENTARY MATERIAL [file 13244_2025_1980_MOESM1_ESM.pdf]

CT-based AI framework leveraging multi-scale features for predicting pathological grade and Ki67 index in clear cell renal cell carcinoma: a multicenter study

ELECTRONIC SUPPLEMENTARY MATERIAL

Supplementary Figure

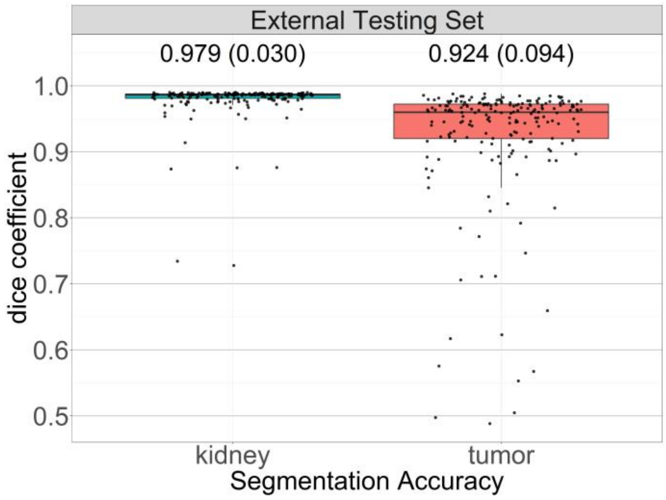

Fig.S1 A dice coefficient of predictive segmentation (kidney and tumor) in external testing dataset. The mean value and standard deviation are shown.

Supplementary Tables

Table S1 Comprehensive parameters regarding the acquisition of CT images for each cohort.

Center	Tube voltage (kV)	Tube current (mA)	Matrix	Slice thickness (mm)	Pitch
Cohort 1	N/A	N/A	512 × 512	2 or 5	N/A
Cohort 2	120	220	512 × 512	1 or 0.625	0.83
Cohort 3	120	280	512 × 512	0.625	0.89
Cohort 4	120	360	512 × 512	1 or 2	0.98
Cohort 5	120	200	512 × 512	1 or 0.625	0.92
Cohort 6	120	340	512 × 512	0.625	0.96
Cohort 7	120	200	512 × 512	0.625	0.87

Table S2. Predictive performances of the pathological grade and Ki67 index classifications, in the internal and external cohorts

	**Pathological Grade**		**Ki67 Index**	
	**Internal Cohort**	**External Cohort**	**Internal Cohort**	**External Cohort**
**Sensitivity**	0.88(0.74-0.96)	0.81(0.64-0.93)	0.82(0.66-0.93)	0.84(0.65-0.95)
**Specificity**	0.70(0.59-0.77)	0.72(0.65-0.78)	0.84(0.75-0.90)	0.71(0.64-0.77)
**Accuracy**	0.74(0.66-0.81)	0.73(0.67-0.79)	0.83(0.76-0.89)	0.73(0.66-0.79)

Supplementary Method

Segmentation model and classifier algorithm

In this study, the construction of an automated semantic segmentation model for CT images adopts the 3D U-Net framework from deep learning. U-Net is a convolutional neural network model widely used in medical image segmentation, comprising a down-sampling path and an up-sampling path. The down-sampling path consists of a series of convolutional and pooling layers to extract features and reduce image dimensions. These layers gradually halve the size of the feature maps until obtaining small-sized feature maps. The up-sampling path includes deconvolutional layers and convolutional layers to restore the size of the feature maps to the input image's dimensions and perform segmentation. The structure of the 3D U-Net model is illustrated in Fig.S1.

We utilized a dataset comprising 857 internally queued CT images as the training set. Each image was meticulously annotated with ROI for the training of the kidney/tumor segmentation model. To evaluate the model's generalization capabilities, an external queue consisting of 216 CT images was allocated as the designated test set. Throughout the training regimen, diverse image augmentation techniques, including flipping, rotation, grayscale adjustments, and Gaussian blur, were judiciously applied. These techniques were implemented with the objective of enriching the diversity of training samples while concurrently mitigating the potential risk of model overfitting. Moreover, a five-fold cross-validation methodology was incorporated during the model training process. The optimal training parameters were meticulously chosen from 1000 iterations to be subsequently utilized in automated segmentation predictions.

Throughout the process of training the neural network, we amalgamate the cross-entropy loss function with the DICE segmentation loss to formulate the ultimate loss function. This composite loss function is employed to calculate the network's gradient and subsequently update the network parameters through the backpropagation algorithm. The nomenclature "DICE loss" is derived from the DICE coefficient, a metric designed to gauge the similarity between two samples of images. A heightened DICE coefficient signifies a more substantial similarity between the two image samples. The mathematical expression is as follows:

$$DICE = \frac{2|X \cap Y|}{|X| + |Y|}$$

In this context, |X∩Y| represents the count of intersecting elements between two samples X and Y. The mathematical expression for the DICE loss function is given by:

$$DICE\ loss = 1 - DICE = 1 - \frac{2|X \cap Y|}{|X| + |Y|}$$
